# Is it good to be bad? An evolutionary analysis of the adaptive potential of psychopathic traits

**DOI:** 10.1017/ehs.2022.36

**Published:** 2022-08-11

**Authors:** Ioana Ene, Keri Ka-Yee Wong, Gul Deniz Salali

**Affiliations:** 1 Department of Anthropology, University College London, 14 Taviton Street, London WC1H 0BW, UK; 2 Department of Psychology and Human Development, University College London, 25 Woburn Square, London WC1H 0AA, UK

**Keywords:** Psychopathy, gene–environment interactions, fitness, frequency-dependent selection, adaptive calibration model, evolutionary medicine

## Abstract

Although psychopathy is widely conceptualised as a mental disorder, some researchers question the maladaptive nature of psychopathy, and argue that it might be advantageous from an evolutionary point of view. According to this view, psychopathy can be seen as an evolutionary adaptative strategy that relies on deception and manipulation to gain short-term reproductive benefits. Psychopathy is also identified as a fast life strategy in response to early life stress and an adaptation to harsh environments. This paper investigates the evidence that psychopathic traits are adaptive, while also addressing the limitations of current evolutionary models of psychopathy based on frequency-dependent selection and life history theory. We review recent studies on the fitness correlates of psychopathy and find that psychopathic traits present potential adaptive trade-offs between fertility and mortality, and offspring quantity and quality. On a proximate level, individual differences in stress reactivity and environmental risk factors in early development predispose to psychopathy through gene–environment interactions. We propose that environmental, developmental, social and cultural factors can mediate the relationship between psychopathic traits and fitness and therefore should be considered to make accurate predictions on the adaptive potential of psychopathy. We end by outlining gaps in the literature and making recommendations for future evolutionary research on psychopathy.

## Graphical abstract

**Figure:**
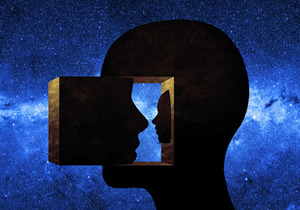

**Social media summary:** Can psychopathic traits be adaptive? Evolutionary perspectives should consider developmental, social, and cultural factors.

## 1. Introduction

Psychopathy is an inherently fascinating and controversial topic. This fascination is particularly evident when looking at the myriad of movies and true crime documentaries which have a psychopathic protagonist (Hesse, 2009). In turn, popular culture portrayals have created a stereotyped and romanticised public perception of psychopaths as mad men, serial killers and unscrupulous geniuses (Skeem et al., 2011). Beyond this public fascination, psychopathy has important legal and forensic implications as it is disproportionately represented in the criminal justice system. While studies that quantify the prevalence of psychopathy are limited, some studies estimate that individuals officially diagnosed with this condition comprise 20–30% of prison populations, 15% of forensic psychiatric patients and around 1–3% of the total population (Coid et al., 2009a; Hare et al., 1991; Johnson, 2019). Gender may also play a role in the expression of psychopathy as that prevalence is at least twice as high in men compared with in women (Coid et al., 2009a, 2009b; Lilienfeld et al., 2014; Johnson, 2019). Moreover, longitudinal studies also show that psychopathic traits are expressed early in life, and that they remain stable across the lifetime (Blonigen et al., 2006; Fanti & Kimonis, 2017; Forsman et al., 2008; Hawes et al., 2017, 2018; Hemphälä et al., 2015; Hyde et al., 2013; Loney et al., 2007; Lynam et al., 2007).

Among the prison population individuals with psychopathy account for a large proportion of severe and violent crimes (Brown & Forth, 1997; Harris et al., 2001; Hart, 1998). This is probably a consequence of the lack of empathy, guilt and remorse in psychopaths, which are necessary for the inhibition of antisocial and violent tendencies (Hare et al., 2012). Not only does psychopathy predispose to violence and crime, but it is also highly predictive of violent recidivism (Hare, 2002; Hemphill et al., 1998; Salekin et al., 1996), and is particularly unresponsive to treatment (Harris & Rice, 2006; Salekin et al., 2010). Despite the media attention and its legal and ethical implications, there is an overwhelming lack of understanding and consensus regarding the biological, developmental and evolutionary roots of psychopathy (Glenn et al., 2011; Gonzalez-Tapia et al., 2017), and hence treatment options remain limited. In this paper, we aim to fill this gap and critically review the theories on the adaptive function of psychopathy.

Psychopathy is a personality disorder broadly defined by shallow emotions, lack of empathy or remorse, deceptiveness, grandiosity, impulsivity, irresponsibility and chronic immoral and antisocial behaviour (Hare & Neumann, 2009). To measure this, the most widely used diagnostic instrument in forensic and clinical settings is the Psychopathy Checklist – Revised (PCL-R) (Hare, 1991), which scores an individual's extent of psychopathy based on 20 traits grouped into two factors: an *interpersonal-affective domain* (factor 1) that encompasses core traits such as callousness and manipulativeness and an *antisocial-lifestyle domain* (factor 2) that entails disinhibition and chronic antisocial behaviour (Hare et al., 1991; Hare & Neumann, 2009; Table 1). Additionally, there are two items which contribute to the total score but do not load on any factors: promiscuous sexual behaviour (item 11) and many short-term marital relationships (item 17) (Hare et al., 2012). Although studies have shown that factors 1 and 2 are moderately correlated and share variance, suggesting some overlap in measurement, these factors are composed of unique traits with different predictive powers (Hunt et al., 2015). The PCL-R rating scale uses semi-structured interviews and case history information to rate each item on the scale and a score of 30 out of 40 is required for a diagnosis of psychopathy (sometimes 25/40 depending on the country; Hare, 2020).

**Table 1.** The Psychopathy Checklist – Revised (PCL-R) items, the most widely accepted clinical measure of psychopathy (after Hare & Neumann, 2009)

PCL-R			
Factor 1		Factor 2	
Interpersonal	Affective	Lifestyle	Antisocial
1. Glibness – superficial charm	6. Lack of remorse or guilt	3. Need for stimulation	10. Poor behavioural control
2. Grandiose sense of self-worth	7. Shallow affect	9. Parasitic lifestyle	12. Early behavioural problems
4. Pathological lying	8. Callous – lack of empathy	13. Lack of realistic, long-term goals	18. Juvenile delinquency
5. Conning	16. Failure to accept responsibility	14. Impulsivity	19. Revocation of conditional release
—	—	15. Irresponsibility	20. Criminal versatility

While the PCL-R is an important tool for the diagnosis of psychopathy in clinical and forensic settings, there is a lot of debate surrounding the classification of psychopathy and the validity of a strict classification of psychopaths and non-psychopaths in the general population (Edens et al., 2006; Hare, 2020; Wright, 2009). The current view is that psychopathy is dimensional, rather than categorical: psychopathic traits exist on a continuum of severity in the population (Edens et al., 2006; Guay et al., 2007; Miller et al., 2001; Walters et al., 2007a, 2007b), and this has been argued for all personality disorders (Tyrer, 2012; Zimmerman et al., 2011). Thus ‘psychopaths’ are at the extreme end of psychopathic personality which can be found at subclinical levels in the general population, and do not represent a separate class of individuals per se (Edens et al., 2006). While the PCL-R cut-off point for psychopathy is useful for forensic, clinical and legal purposes where such categorisation may serve a function for recommendations and treatment, we should consider all people to be more or less psychopathic. The dimensional nature of psychopathy has important implications when looking at its adaptive potential, as we will discuss later on, because different psychopathic domains (interpersonal, affective, antisocial, lifestyle) can have different fitness outcomes. Thus, in the general population psychopathy is measured with self-report scales modelled after the PCL-R such as the Self-Report Psychopathy scale (Williams et al., 2003), the Psychopathic Personality Inventory (Lilienfeld & Andrews, 1996) and the Levenson Self-Report Psychopathy Scale (Levenson et al., 1995). Another useful area of research for psychopathy comes from studies in psychology on dark personality traits, which include psychopathic components. One of the most commonly used measures is the Dark Triad, which is a construct of three non-pathological, socially aversive personalities: Machiavellism, subclinical narcissism and subclinical psychopathy (Paulhus & Williams 2002).

At a neurological level, psychopaths display impaired emotional processing, characterised by a reduced response to and recognition of fear, pain, shock and happiness (Aniskiewicz, 1979; Blair et al., 1997; Dawel et al., 2012; Rothemund et al., 2012). In addition, psychopathy has been linked to attentional deficits which make psychopaths less able to focus on emotional stimuli (Baskin-Sommers et al., 2011; Hiatt et al., 2004; Newman et al., 2010). These atypical emotional and attentional processes are often, but not always, accompanied by functional ‘deficits’ in areas of the brain that are either located in the limbic system or adjacent to it (amygdala, prefrontal cortex, hippocampus, temporal cortex and anterior and posterior cingulate; Glenn & Raine, 2008; Harenski et al., 2010; Vien & Beech, 2006). Nonetheless, there are debates regarding whether the differences between psychopathic and non-psychopathic brains represent ‘dysfunctions’, and whether they are a causal factor in the disorder (Hare et al., 2012; Nadelhoffer & Sinnott-Armstrong, 2013).

While traditionally conceived as a mental disorder, this idea has been challenged. Several researchers argue that psychopathy does not seem to fulfil the criteria for a ‘dysfunction’ in a biological sense (Barr &d Quinsey, 2004; Jurjako, 2019; Leedom & Almas, 2012; Lilienfeld & Marino, 1995; Kendell, 2002; Krupp et al., 2013). The debate is generally centred around divergent interpretations of Wakefield's (1992: 373) sociobiological definition of a harmful dysfunction, which states that ‘harmful is a value term based on social norms, and dysfunction is a scientific term referring to the failure of a mental mechanism to perform a natural function for which it was designed by evolution’. Similar approaches have been applied to explain other mental disorders and maladaptive traits where psychopathology is seen either as an adaptive response to the environment or as a by-product of adaptations involved in evolutionary trade-offs, and not as a biological dysfunction (Hunt & Jaeggi, 2022; Nesse, 2004; Nesse & Stein, 2019; Raihani & Bell, 2019). Arguments can be broadly divided between those who argue that neurological markers and antisocial tendencies are indicative of a dysfunction (Leedom & Almas, 2012; Nadelhoffer & Sinnott-Armstrong, 2013) and those who state that neurological differences do not necessarily imply a dysfunction and that the evidence that psychopathy is disadvantageous at an individual level is weak (Barr & Quinsey, 2004; Jurjako, 2019; Krupp et al., 2013; Wiebe, 2004). The self-centred, coercive and manipulative nature of psychopathy has led several evolutionary scientists to question the maladaptive nature of psychopathy (Barr & Quinsey 2004; Brazil et al., 2019; da Silva et al., 2015; Del Giudice et al., 2011; Mealey, 1995a; Wiebe, 2004). They have argued that psychopathy could have evolved owing to selective pressures and might instead be an adaptation.

In this paper we will review the evolutionary explanations for psychopathy and address the question whether psychopathy is adaptive. We will go over the explanations based on frequency-dependent selection and life history theory and highlight the empirical support for and limitations of those explanations (Section 2). We will then review the studies examining the correlation between psychopathic traits and fitness proxies that concern reproduction and survival (Section 3). Our main argument shall be that certain environmental factors, such as stress experienced early in life, the nature of social interactions (e.g. the behaviour of the receiver) and social structure and cultural norms can mediate the relationship between psychopathy and fitness. Therefore, assessing the adaptiveness of psychopathic traits requires an interdisciplinary approach combining evolutionary, developmental, anthropological and psychological studies. We finish by proposing future research avenues based on the current gaps in the literature (Section 4).

## 2. Evolutionary explanations of psychopathy

### 2.1. Frequency-dependent selection

The facts that psychopathy has been reported across several cultures (although we will discuss this further in Section 3.2), is moderately heritable and stable across the lifetime, and often serves the needs and interests of the individual have led many researchers to explore the possibility that psychopathic traits are the result of a functional evolutionary strategy (Wiebe, 2004). In evolutionary research, psychopathy is most often discussed in relation to frequency-dependent selection (Glenn et al., 2011; Mealey, 1995a, 1995b; Book et al., 2019). Under the frequency-dependent selection model, the advantage of certain behaviours and traits present in a population depends on their frequency: there is a predominant cooperative strategy which is beneficial at high frequencies, accompanied by less common, non-cooperative strategies which are only beneficial when the individuals displaying it are low in number (Ayala & Campbell, 1974). As a highly social species, our fitness depends on cooperation with other individuals and most humans are considered prosocial or cooperative strategists (Wiebe, 2012). Therefore, psychopaths who display impaired social cognition and emotions are regarded as antisocial strategists who instead rely on exploiting others at their own advantage (Gervais et al., 2013). Their inability to relate to and recognise fear in others could make them impervious to threat which in turn can facilitate such exploitative strategies (Book et al., 2020).

The assumption that psychopathy is a cheater strategy maintained under frequency-dependence generates several predictions on the behaviour and prevalence of psychopaths. Firstly, psychopathy is found at low levels in the population (approx. 1–3%, based on PCL-R diagnosis; Hare, 1996; Johnson, 2019), and this seems to fit the predictions of the frequency-dependent selection. At a high frequency, cheaters become more easily detectable, and are more likely to encounter other cheaters, which undermines the effectiveness of this strategy (Brazil et al., 2019). Secondly, if psychopathy represents an antisocial strategy, then we would expect psychopaths to possess behavioural strategies aimed at exploiting others for their own benefit. Indeed, psychopathic traits are associated with both a desire to deceive others (Seto et al., 1997) and the ability to deceive others efficiently by appearing genuine during deceptive interactions (Book et al., 2015; Porter et al., 2011; Brazil et al., 2021). Psychopaths are also more likely to engage in uncooperative strategies in social dilemma studies in university (Curry et al., 2011; Gervais et al., 2013), as well as prison samples (Mokros et al., 2008).

While the idea of psychopaths as cheaters has received some support, there are some limitations to this theory that we would like to address. Frequency-dependent selection is a model used to explain behavioural variance and the co-existence of different behavioural morphs as a result of stable, obligate, genetically influenced strategies employed by certain non-human species (Jones et al., 2015; Lalumière et al., 2008). One well-known example is the alternative male phenotypes in bluegill sunfish: larger, parental males who invest in growth and reproduction, and smaller ‘sneaker’ males who engage in quick and transient copulations (Gross & Charnov, 1980). While cheater phenotypes have been documented in species such as insects and fish, currently the presence of equivalent strategies in highly social species has not received enough empirical support (Riehl & Frederickson, 2016). The specific genetic mechanisms that maintain variance in personality traits in humans, and in particular the role of balancing selection (which refers to adaptive forces that maintain genetic variation in populations and includes frequency-dependent selection) have not been firmly established yet (Penke & Jokela, 2016). If psychopathy is maintained in populations under frequency-dependent selection, then we should expect it to have a strong genetic component (Fitzpatrick et al., 2007; Trotter & Spencer, 2007). However, recent evidence shows that psychopathy is the result of multiple genetic and environmental factors which interact in dynamic ways (for a comprehensive review on this topic see Maung, 2021). Psychopathy might have a genetic component, as shown by heritability studies that estimate that 40–60% of the variance in psychopathy can be explained by genetic factors (Bezdjian et al., 2011; Blonigen et al., 2003, 2005; Glenn & Raine, 2014: 23; Johansson et al., 2008; Larsson et al., 2006; Taylor et al., 2003; Vernon et al., 2008; Viding et al., 2005). However, genes most likely confer vulnerability to developing psychopathy, rather than causing it directly (Viding et al., 2014).

Moreover, explanations of psychopathy based on frequency-dependent selection assume that psychopaths are a distinct class of individuals that can be separated from non-psychopaths. However, the dimensional nature of psychopathic traits questions the existence of a specific exploitative strategy of this kind. A critique of the view of specific strategies is that humans have been shaped by natural selection to respond more flexibly to the environment, rather than to rely on fixed strategies (Buss & Greiling, 1999; Glenn et al., 2011). As psychopathic traits vary on a continuum of severity in populations, and psychopathic traits are a result of an interplay between multiple factors in development, we believe that a more nuanced approach is required to explain whether and how psychopathy is adaptive. This would require a consideration of how psychopathic traits develop, which can ultimately elucidate how the fitness benefits of psychopathy are delivered. In the next section, we will review a different evolutionary explanation for psychopathy that takes into account the variation in early life environment and is based on life history theory.

### 2.2. Life history theory

#### 2.2.1. Life history theory in a nutshell

Life history traits are traits of an organism that concern growth, reproduction and survival: the age and size at maturation, number and size of offspring, age at first reproduction, the overall lifespan, etc. Life history theory (LHT) explains inter-species variation in those traits as a result of environmental variation (in mortality risk) and predicts trade-offs between different fitness components (e.g. reproduction and somatic maintenance, quality and quantity of offspring; Flatt & Heyland, 2011; Stearns, 1976). Accordingly, species can be classified into fast, ‘r-selected’ strategists (high growth rate, fecundity and mortality) and slow, ‘K-selected’ (slow growth rate, fewer offspring, longer lifespan) strategists (MacArthur & Wilson, 1967). Some scholars have argued that individuals can vary on this slow–fast continuum within a species, and this premise has been applied to certain human traits and behaviours (for critical reviews see Nettle & Frankenhuis, 2020; Sear, 2020). Specifically, a harsher and more unpredictable environment (i.e. higher extrinsic mortality) early in life is predicted to favour behavioural traits associated with fast life strategies (Brumbach et al., 2009). Following from this view, psychopathy represents a fast life history strategy focused on investment in mating effort over parenting effort (Barr & Quinsey, 2004; Jonason et al., 2009, 2010).

#### 2.2.2. Early life environment, stress management and psychopathy

Our species possesses a high degree of developmental plasticity, which enables us to react to external influences from our environment, especially in early life, leading to changes in our brain, anatomy and physiology (Frankenhuis & Amir, 2022). This is evolutionarily advantageous because it means that we can respond to the environment flexibly and adapt to adversities. At a molecular level this occurs through epigenetic processes which alter genetic expression without changing genetic sequences, and this in turn irreversibly alters the development of an individual (Lea et al., 2017). Recent evidence shows that genes and the environment are interdependent in predicting individual susceptibility to psychopathy (Maung, 2021). Accordingly, individuals with an inherited risk might develop the condition only in the presence of environmental triggers, and vice-versa.

Longitudinal studies have linked psychopathy in adulthood to several detrimental factors in childhood, such as low neighbourhood socioeconomic status, parental criminal record, mother's low level of education and mother's young age of pregnancy, factors thought to contribute to non-optimal parenting (Bamvita et al., 2017; Farrington et al., 2006; Piquero et al., 2012). This correlation between psychopathic traits and early stress seems to be moderated by genetic and developmental factors. For instance, low socioeconomic status was associated with callous–unemotional traits only in youth with a homozygous genotype for the serotonin transporter protein gene (SLC6A4), which is associated with reduced threat and stress responsivity (Sadeh et al., 2010). In a longitudinal study, Beaver et al. (2015) found that infants with an easy temperament were the most at risk of presenting affective psychopathic traits in adolescence if they experienced low parental sensitivity, while being the least at risk if they experienced high parental sensitivity. The researchers further argued that individuals might have differential susceptibility to developing psychopathy, whereby infants who are more sensitive to the environment might be especially susceptible to external conditions for better or worse (Ellis et al., 2011). Callous–unemotional (CU) traits are a constellation of traits related to lack of empathy, guilt, low empathy and interpersonal callousness, which are a hallmark of psychopathy in youth (Forsman et al., 2008). Willoughby et al. (2013) found that harsh-intrusive parenting in infancy predicted callous–unemotional traits at age 3, and that this relationship was stronger for children with a polymorphism of the brain-derived neurotropic factor (BDNF) gene, which is involved in processing fear and learning from punishment. Another study is reflective of how the environment can affect biological predisposition: in an adoption cohort of 561 families, the biological mother's self-reported antisocial behaviour predicted children's CU traits at 27 months despite not having contact with the children (pointing to a genetic/inherited bias), but the study also showed that the adoptive mother's positive reinforcement buffered the prenatal risk and protected against CU traits (Hyde et al., 2016). Other studies have further validated the role of both heritable risk and protective factors: the biological mother's fearlessness predicted CU traits in children, while the adoptive mother's positive parenting buffered against this inherited bias (Waller et al., 2017).

**Figure 1.**
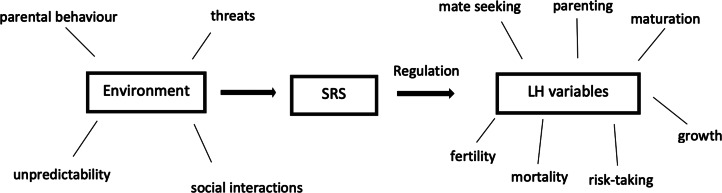
Structure of the stress response system and its regulatory processes. The stress response system encodes information from the local environment which feeds back into the development of life history traits (after Del Giudice et al., 2011).

A recently proposed model could explain this role of the environment in facilitating psychopathic traits, namely the Adaptive Calibration Model (ACM; Del Giudice et al., 2011). The ACM uses an evolutionary-developmental framework to explain individual differences in stress responsivity and how they affect behaviour. According to the ACM, the calibration of an individual's stress-response system, composed of the autonomic nervous system and the hypothalamic–pituitary–adrenal axis, influences the development of life history-related traits such as mating, risk-taking and parenting (Del Giudice et al., 2011). The pattern an individual adopts depends on both genetic predisposition and environmental conditions early in life, which can affect the body's response to stress. Harsh and threating environments can cause a frequent activation of the stress-response system, which can alter gene expression, which in turn affects hormone levels and behavioural outcomes (Figure 1). Over time this can lead to the adoption of an unemotional pattern characterised by low stress responsivity via low stress-response system activity. Arguably, this can be advantageous because it protects individuals from stress, while also facilitating a fast life history strategy which is beneficial in ‘dangerous’ environments (Del Giudice et al., 2011; Ellis & Del Giudice, 2019). This is achieved because individuals become insensitive to danger and negative social feedback (disproval, shame, etc.), which enables competitive risk taking, antisocial behaviour and impulsivity. Moreover, this pattern is predicted to be male biased because a fast strategy focused on risk-taking and mating effort may be more beneficial for males (Del Giudice et al., 2011), based on the sex differences in the consequences of mating and parenting efforts (Trivers, 1972; Kokko and Jennions, 2008).

Psychopathic traits are not only correlated with stressful conditions in early life, but also seem to protect against stress later in life. There is evidence that psychopathic traits protect against posttraumatic stress through emotional numbing (Anestis et al., 2017; Hicks et al., 2010; Kerig et al., 2012; Willemsen et al., 2012). In a student sample, affective psychopathic traits were negatively correlated with indicators of emotional distress (depression, anxiety and stress; Međedović et al., 2018). Similarly, in male inmates interpersonal-affective traits were correlated with lower rates of anxiety (Sandvik et al., 2015). These findings show that psychopathy can act as a coping mechanism for emotional distress, while also reducing mental illness associated with it. These observations fit the predictions of the ACM that the unemotional and remorseless aspect of psychopathic traits should be advantageous in adverse environments because it buffers against the detrimental effect of long-term stress (Del Giudice et al., 2011).

The behavioural traits associated with life history strategies involve risk-taking, parenting and mating behaviours, and some scholars suggested that psychopaths adopt a fast life history strategy focused on investment in mating effort over parenting effort (Barr & Quinsey, 2004; Jonason et al., 2009, 2010). This prediction has received some empirical support. Jonason et al. (2010) used the Mini-K, a 20-item measure of life history strategy, to show that psychopathic traits are correlated with life history characteristics such as risk-taking, future discounting, and social, familial and sexual relationships that are representative of a fast strategy. It is important to note here that there have been some criticisms of the application of LHT to psychology. In their reviews both Sear (2020) and Nettle and Frankenhuis (2020) have argued that LHT applied to psychology significantly differs from the life history research in evolutionary biology. While the latter relies on testable mathematical models quantifying variations in strategies according to ecological differences at population and species level, LHT as applied to human psychology lacks theoretical formalisation and relies on intuitive reasoning as to why certain traits might facilitate a specific strategy (Nettle & Frankenhuis, 2020). Moreover, psychological research using LHT is often limited to Western, educated populations, which limits our understanding of the variation in life history strategies under different environments (Sear, 2020). Resolving these issues and integrating evolutionary and psychological models of LHT is required to appropriately assess whether psychopathy represents a fast life history strategy in a strict sense.

## 3. Is psychopathy adaptive?

### 3.1. Psychopathic traits are involved in adaptive trade-offs

The most common definition of evolutionary fitness is based on the probability of an individual passing their genes to the next generation, which is referred to as ‘reproductive fitness’ (Hamilton, 1964), although current fitness definitions remain contentious (Kuzenkov & Morozov, 2019; Orr, 2009). More accurately, we can extend the definition of fitness to represent the survival of a genetic lineage which depends not only on individual reproduction and survival, but also on the survival of those carrying one's genes, termed ‘inclusive fitness’ (Akçay & Cleve, 2016; West & Gardner, 2013). Therefore, considerations of how individuals interact with their close social group and kin are important for assessments of evolutionary fitness.

Both the frequency-dependent selection based and LHT-based evolutionary explanations of psychopathy predict it to be fitness-maximising under certain conditions, ultimately enhancing survival and reproduction. The frequency-dependent selection model predicts psychopathic traits to be adaptive when they are expressed in low frequencies, while LHT-based explanations predict those traits to be adaptive under certain developmental contexts. What evidence do we have about psychopathic traits and their fitness correlates? In this section, we will review the primary research on psychopathic traits and fitness proxies, behavioural tendencies that can influence reproduction and survival, as well as some measurable fitness outcomes. Table 2 summarises the reviewed studies and their main findings. It is worth mentioning that these fitness proxies may not directly correspond to actual fitness, a limitation which we will discuss below.

**Table 2.** Summary of the literature on the fitness correlates of psychopathic traits and their potential relevance for reproduction and/or survival

Authors	Design	Sample	Potential relevance for reproduction and/or survival	Summary
Dion et al., 1997	Self-report questionnaire	Community sample of divorced parents	Low parental investment	Non-custodial parents high on psychopathic deviance were less likely to offer child-support payments than were those lower on psychopathy, controlling for the parent's employment status
Kirkman, 2005	Qualitative study based on interviews and written accounts of women who dated psychopathic men (as rated by the women)	A group of women who have been in long-term relationships with men who displayed psychopathic traits	Reduced long-term partner potential and low parental investment	Women reported a range of abusive behaviours from their psychopathic partners including emotional abuse, psychological torture, infidelities, lying, victimisation, economic abuse (parasitising on the woman's resources), physical assault and controlling behaviours (e.g. preventing the woman from meeting with family and friends). The women also reported that their partners would often emotionally abuse their children by lying, bullying and ignoring them, while also failing to provide for them, breaking promises, hiding food from them and destroying their toys
Harris et al., 2007	Analysis of clinical file materials	Male sex offenders	Increased mating effort	Psychopathy strongly associated with coercive and precocious sexuality
Jonason et al., 2009	Self-report questionnaire	Undergraduates	Increased mating effort	Students higher in dark traits, and especially psychopathy, reported having more sex partners, a greater preference for short-term mates and higher sociosexuality (i.e. a tendency towards unrestricted sexual relationships), and this correlation was stronger for men
Fulton et al., 2010	Self-report questionnaire	Undergraduates	Increased mating effort	Lifestyle-antisocial traits predicted risky sexual behaviours (e.g. multiple partners, casual sex with unknown partners, sex without a condom and using alcohol or drugs prior to or during sex) in men and women, whereas affective-interpersonal traits only predicted it in men
Visser et al., 2010	Self-report questionnaire and other-ratings	Undergraduates	Increased mating effort	Psychopathy associated with early sexual behaviour, promiscuity and affairs in both men and women. In men psychopathy was also associated with high self- and other-rated attractiveness, low appearance anxiety and low body shame. In women, psychopathy was associated with low self-esteem and high body shame
Kastner and Sellbom, 2012	Self-report questionnaire	Undergraduates	Increased mating effort	Psychopathic traits were correlated with hypersexuality (sexual sensation seeking, compulsivity, excitation, disinhibition, risky sexual behaviour). In addition, individuals scoring high on both facets of psychopathy had a higher propensity for risky sexual behaviour than those scoring high in only one of them
Krupp et al., 2012	Data from a review of 400 case files			
	Violent male offenders	Nepotistic tendencies	Psychopathy negatively correlated with victim–offender relatedness	
Neumann et al., 2012	Archival data on demographic indices from the UN Human Development Report 2005	Mixed student and community sample	Increased fertility, but increased offspring mortality	In women the interpersonal facet of psychopathy, and to a lesser but nonetheless significant degree the antisocial domain, were positively correlated with regional measures of fertility, as well as maternal and infant mortality
Holtzman and Strube, 2013	Other participants rated photographs of study subjects	Undergraduates	Increased mating effort	Psychopathy associated with effective adornment – i.e. dressing and presenting oneself in ways that increase physical attractiveness
Leedom et al., 2013	Qualitative study based on memoirs	Children of parents with psychopathic traits (as described by the children)	Low parental investment	Psychopathic parents displayed a wide range of poor parenting behaviours: picking favourite children and treating them favourably; acting affectionately only to secure power and control over children; manipulating children if they disobeyed them; not showing concern for the wellbeing and health of their children; a mix of loving and abusive behaviours; emotional abuse was predominant and, in some cases, physical abuse as well
Beaver et al., 2014a	Self-report questionnaire	Community sample	Increased morbidity	Higher psychopathy scores predicted a significant reduction in general health and an increase in chronic (diabetes, high blood pressure, high cholesterol, etc.) and neurological (ADHD, anxiety, depression, etc.) disorders
Beaver et al., 2014b	Self-report questionnaire	Community sample of parents	Low parental investment	Both males and females higher on psychopathic personality traits reported more negative parenting quality on average even after controlling for child temperament effects
Jonason et al., 2015	Self-report questionnaire	Undergraduates and high-school students	Increased morbidity	Higher scores of psychopathy were associated with a lower life expectancy based on a multitude of health metrics and behaviours – body mass index, hereditary disease, stress, exercise, toxin consumption and living habits
Visser et al., 2015	Self-report questionnaire	Undergraduates	Increased mating effort	Psychopathy predicted sexual fantasies related to multiple, anonymous and uncommitted partners, as well as actual engagement in similar real-life sexual behaviours
Hudek-Knežević et al., 2016	Self-report questionnaire	Community sample	Increased morbidity	Psychopathy was a negative predictor of protective health behaviours and consistently increased the risk of digestive diseases, tobacco use and injuries
Jonason and Lavertu, 2017	Self-report questionnaire	Community sample of women	Increased morbidity and potential decreased fecundity	Women high in psychopathy reported more lifetime sexual health problems, pain associated with sexual health and miscarriages
Međedović et al., 2017	Self-report questionnaire	Male convicts	Mixed results for fertility	Interpersonal features are associated with higher numbers of children, whereas the opposite effect was noticed for affective and lifestyle traits. Affective traits elevated fertility only for individuals raised in families with substance abuse
Monteiro et al., 2017	Self-report questionnaire	Undergraduates	Increased mating effort	Psychopathy was correlated to higher frequencies of self-promoting mate attraction behaviours (i.e. making good first impressions, using flirtation and physical contact, etc.) in a short-term mating context in men (but not women)
Carter et al., 2018	Self-report questionnaire	Community sample	Reduced fertility	For both men and women psychopathy was a negative predictor of lifetime offspring (age-adjusted)
Međedović, 2019a	Self-report questionnaire	Community sample (post-reproductive)	Mixed results for fertility and offspring mortality	Interpersonal features positively predicted the lifetime number of children and negatively predicted the number of grandchildren and physical illness. Lifestyle traits positively predicted physical illness in offspring
Međedović, 2019b	Self-report questionnaire	Community sample of parents	Increased mating effort and low parental investment	Psychopathy associated with earlier onset of sexual activity, shorter partner relationships, higher mate seeking and reduced care for children
Međedović and Petrović, 2019	Self-report questionnaire	Community sample (post-reproductive)	Increased fertility and low parental investment	All psychopathic traits assessed (emotional coldness, deceitfulness, recklessness) correlated positively with reproductive success. However, fertility is predicted primarily by emotional coldness. On the other hand, psychopathic traits were negatively correlated to parental investment
Smith et al., 2019	Self-report questionnaire	Undergraduates	Increased mating effort and reduced long-term commitment	Psychopathy associated with motivation to have sex for stress relief and less partner-focused motivations
Brazil and Forth, 2020	Multimethod (self-report survey, other-rated videos)	Male undergraduates	Increased mating effort	Psychopathy associated with higher sociosexuality, and higher desirability ratings from women on video recordings in a dating scenario. Lifestyle traits were the strongest predictor of desirability ratings from women
Lyons et al., 2020	Self-report questionnaire	Community sample	Potential for low parental investment	Psychopathy positively correlated to a desire for low parental care in prospective partners
Međedović and Kujačić, 2020	Data from prison files, medical records, self-report measures	Male convicts	Increased morbidity	Factor 2 traits (lifestyle and antisocial) are significantly correlated to diminished physical and mental health. Factor 1 traits were unrelated to or moderately associated with improved health.

Although there are a few studies investigating the correlation between psychopathic traits and direct measures of fitness (e.g. lifetime number of children and number of grandchildren), most studies use fitness proxies such as mating and parenting behaviour (Table 2). Psychopathic traits have been associated with desires, motivations and behaviours that reflect heightened sexuality, which could influence reproductive success. For example, they are linked to promiscuous sexual fantasies and behaviours (Jonason et al., 2009; Visser et al., 2010, 2015), an early sex life (Harris et al., 2007; Međedović, 2019b; Visser et al., 2010), sexual risk-taking and sensation-seeking (Fulton et al., 2010; Kastner & Sellbom, 2012), and unrestricted sexual relationships (Jonason et al., 2009). Psychopathic individuals not only seek out short-term relations, but also potentially possess the ability to attract mates through favourable impressions (Brazil & Forth, 2020; Holtzman & Strube, 2013; Monteiro et al., 2017; Visser et al., 2010). These observations suggest that psychopathic traits not only influence the desire for promiscuity and short-term relationships, but potentially also reduce the inhibition of those behaviours.

To test the predictions from the LHT, several studies investigated the associations between early life environment, psychopathic traits and fitness proxies. In a community sample, individuals higher in the affective component of psychopathy which captures a lack of empathy had an especially strong relationship between childhood environmental harshness (family dysfunction, poverty) and high mate seeking, short partner relationships and lower parental effort (Međedović, 2019b). Likewise, the same study group found that in male convicts, affective traits promoted reproductive success in harsh environments: individuals with affective traits had a higher number of children if raised in families with parental substance abuse than if they were raised in families without substance abuse (Međedović et al., 2017). Thus, a harsh environment can mediate the relationship between psychopathy and fitness and serve as an adaptive response to stress.

Meanwhile, psychopathy could have detrimental effects on long-term relationships as it is often correlated with a lack of investment in partners (Međedović, 2019b; Smith et al. 2019) and abuse in relationships (Kirkman, 2005). Parents with higher scores of psychopathy show lower parental investment and more negative parenting in self-reported (Beaver et al., 2014b; Međedović, 2019b) and offspring-rated questionnaires (Međedović & Petrović, 2019), as well as in qualitative studies (Kirkman, 2005; Leedom et al., 2013). Additionally, psychopathy might negatively influence whether individuals support their children financially (Dion et al., 1997), while also making them more likely to favour partners that also display low parental care (Lyons et al., 2020). The findings summarised in Table 2 highlight potential adaptive trade-offs between fertility and mortality, as well as offspring quantity and quality. In terms of fertility, findings are inconsistent with studies showing both positive (Međedović & Petrović, 2019; Neumann et al., 2012) and negative correlations (Carter et al., 2018), while others suggest that only interpersonal traits of psychopathy positively predict fertility (Međedović, 2019a; Međedović et al., 2017). However, the use of different samples (men vs. women; reproductive vs. post-reproductive individuals) could be partially responsible for these inconsistent findings. On the other hand, psychopathic scores, especially in the lifestyle-antisocial domain, predict higher individual and offspring mortality and morbidity (Beaver et al., 2014a; Hudek-Knežević et al., 2016; Jonason et al., 2015; Međedović & Kujačić, 2020; Neumann et al., 2012), as well a potential decrease in fecundity (Jonason & Lavertu, 2017). This is most likely due to irresponsible, reckless and impulsive behavioural tendencies which can result in negative health outcomes and decreased longevity.

Nevertheless, trade-offs between offspring quantity and quality, and fertility and mortality do not necessarily lead to straightforward predictions on fitness. At present most research is limited to proxies of fitness rather than measurable fitness outcomes, which limits our ability to test the evolutionary predictions. Another point is that research in this area rarely explains why the use of such fitness proxies (e.g. mating and parenting behaviours) is adequate to validate theories. Even if psychopaths engage in multiple sexual relationships, for example, that does not mean that they will have increased fitness as per number of children, compared with individuals who engage in few relationships with long-term commitments. Similarly, a psychopathic parent with lowered parental investment will not have reduced offspring fitness if the other partner compensates or there is adequate resource availability. Moreover, while psychopathic individuals might have abusive parenting styles, there is no considerable evidence that they will endanger the lives of their children. There is only one study to date that looks at nepotistic behaviours in psychopaths. Data from the case files of 289 violent males revealed that psychopathy predicts nepotistic inhibition of violent behaviour: as the PCL-R score increased, the relatedness between the offender and its victim decreased (Krupp et al., 2012). This reflects an inhibition on harming relatives and therefore might increase the inclusive fitness of psychopathic traits.

### 3.2. The importance of cultural context

We should also expect the adaptiveness of psychopathy to depend on the cultural context as different social norms and values will dictate how tolerant people are of psychopathic individuals. Some researchers argue that modern individualistic societies facilitate psychopathic behaviour by encouraging competition and impersonal relationships as opposed to collectivist societies that emphasise altruism and social responsibility (Cooke & Michie, 1999; Jakobwitz & Egan, 2006). Indeed, a recent cross-cultural study showed an association between cultural values associated with individualism and the presentation of psychopathy: psychopathic traits were positively correlated to a value-set of selfishness, a preference for social inequality and a lack of social cohesion, and negatively correlated with collectivist values of group responsibility and integrity (Shou et al., 2021). Empirical observations also show that individuals who score higher on self-report measures of psychopathy place more value on acquiring power, financial success and material possessions, but not on achieving these goals through ambition and achievement (Glenn et al., 2017). Moreover, there is some research that points to the propensity of psychopathic individuals to occupy leadership and high-risk professional roles (Boddy, 2010; Lilienfeld et al. 2014; Howe et al., 2014; Patton et al., 2018). Another insightful area of research is that on ‘corporate psychopaths’, i.e. individuals who score high on psychopathy and tend to occupy power roles in companies (Wellons, 2012). For example, one study showed that in a sample of 203 corporate professionals, 3.9% of the sample presented PCL-R scores of 30 or more (which meets the criteria for a psychopathy diagnosis), which is higher than the 1% seen in the general population (Babiak et al., 2010). These observations are suggestive of the adaptive potential of psychopathic traits in modern societies where occupational prestige can act as a proxy for social ranking. Status-seeking traits (dominance, prestige, etc.) have relevance for fitness in non-egalitarian human societies as they influence resource allocation, mating, cooperation and conflict patterns (Cheng et al., 2010).

In small-scale societies where social norms promote egalitarianism, prestige is not determined by one's individualistic achievements but through prosocial behaviours (Glowacki and Lew-Levy, 2022). For most of human evolutionary history, our species lived by hunting and gathering, hence the key differences between hunter–gatherer and industrialised societies are likely to have profound implications for mental health (for a recent review see Chaudhary & Salali, 2022). One of these key differences lay in the social organisation. Immediate-return hunter–gatherers (who do not store food and are highly mobile) live in campsites of 30–40 people that comprise multiple households (Marlowe, 2005), and the social organisation is characterised by egalitarianism (Woodburn 1982). Decisions are consensus-based, there is no leader or formal social ranking, and there is a heavy emphasis on individual autonomy and rejection of dominant behaviour (Woodburn, 1982). The ultimate driver of this social organisation is the hunting–gathering niche: because successful acquisition of meat is unpredictable, the shortfall in food is compensated for by food-sharing networks (Kaplan et al., 2000; Dyble et al., 2016). Social networks in hunter–gatherers are not only crucial in food sharing but also in childrearing (Page et al., 2017) and the accumulation of medicinal plant knowledge (Salali et al., 2016), which are relevant for individual fitness. Indeed, among the BaYaka hunter–gatherers in Congo, individuals with larger social networks are found to have a higher body mass index and greater fertility (Chaudhary et al., 2016). Individuals who do not cooperate are ostracised (Wiessner, 2005). Thus, for our ancestors, social networks were a matter of life and death, egalitarian group living was the standard and social isolation was rare, with fatal consequences (Chaudhary & Salali, 2022). The strong social networks and egalitarianism of hunter–gatherers could undermine the benefits of antisocial behaviours employed by psychopaths. Psychopaths would also be more likely to acquire an unfavourable reputation in small-scale societies owing to their interconnectedness.

Nonetheless, while we might expect psychopathy to be influenced by cultural factors, empirical observations are yet to establish whether psychopathy rates vary between cultures. There are several studies which confirmed the broad validity of psychopathy and its syndromes across cultures using both PCL-R (Cooke at al., 2005; Fanti et al. 2018; Reale et al., 2022; Shariat et al., 2010; Sullivan et al., 2006; Wilson et al., 2014) and self-report measures (Latzman et al., 2015; Shou et al., 2017) with some subtle differences in total scores and the presentation of specific psychopathic domains. For instance, in a comparison of North American and Iranian psychopathic offenders and patients, while a deficient emotional response was a core characteristic of Iranian psychopaths, interpersonal attributes such as deceitfulness and superficiality did not hold as much predictive power to differentiate between Iranian psychopaths and non-psychopaths (Shariat et al., 2010). The authors suggested that this could be due to the collectivism of Iranian society, whereby individuals are expected to obey societal norms regardless of personal aspirations, and therefore it is more difficult to assess the genuineness of intentions. However, it is important to note that the studies mentioned above were carried out in large-scale, industrialised societies, no studies to date have assessed psychopathy in small-scale societies and few attempt to conduct measurement invariance studies across populations (Mokros et al., 2011).

Currently, the lack of cross-cultural studies undermines the predictive value of adaptive models of psychopathy. There are theoretical expectations as to why the expression of psychopathy should differ in individualistic and impersonal societies vs. collectivist societies according to contrasting societal values (individual success vs. cooperation). Moreover, cultural variations in the expression of specific psychopathic domains of psychopathy (affective, interpersonal, etc.) across cultures could imply that certain psychopathic traits are culture specific. However, such cultural differences could be suggestive of different rates of psychopathy or that psychopathic individuals inhibit their behaviours to comply with societal norms and to avoid ostracism.

## 4. Future directions

So far, we have reviewed evolutionary explanations of psychopathy, the evidence for and shortcomings of those explanations and the studies investigating fitness proxies of psychopathy. Throughout our review we have identified some limitations to the existing data, which in turn have led to the potential future directions below.

Firstly, current evolutionary models of psychopathy often look at proxies of fitness such as increased mating effort or reduced parental investment. Nevertheless, such proxies may not always correspond to reproductive success. As we discussed earlier, the context in which interactions with psychopathic individuals take place can alter the fitness consequences of those interactions (e.g. lack of parental investment can be compensated by cooperative childcare). More studies should investigate fitness outcomes using measures of fertility (total number of children), fecundity (e.g. through measures of reproductive health) and individual and offspring mortality, as these have higher predictive value for reproduction and survival than behavioural proxies only. Additionally, the sample choice is important: post-reproductive individuals might provide the most accurate measure of fitness by assessing lifetime reproductive success (Jones et al., 2015; Orr, 2009). There are also virtually no studies that assess the interactions with kin and nepotistic tendencies of psychopaths. At the moment the lack of data in this area prevents us from making predictions on the inclusive fitness of psychopathic traits, which is a crucial component of evolutionary fitness. Furthermore, an interdisciplinary approach which considers the environmental context and integrates observations from evolutionary, psychological, developmental and anthropological studies is the best way forward. Potentially, this approach would provide insight into the specific factors that confer fitness advantages to psychopathic traits, as well as how they can be maintained by natural selection.

Secondly, theories that conceptualise psychopathy as an adaptive strategy rely on the assumption that psychopathic tendencies to deceive and manipulate automatically translate to actual manipulation taking place. Yet we do not currently have enough data to understand the receiving end of this manipulation, and whether it is indeed effective, or whether there are any social factors that determine who is most susceptible to manipulation by psychopaths. While we understand that genuine interactions of this sort cannot be feasibly assessed, this limitation should be acknowledged in discussions about the advantages of cheating behaviours of psychopaths. Potentially, more interviews or surveys with family members and peers of psychopaths could be insightful on this topic as it might give us an idea about the frequency and efficiency of manipulative tactics employed by psychopathic individuals. In addition, incorporating this data would also help minimise the overreliance on self-report measures, which are predominant in psychopathic literature, especially in studies carried out outside clinical and forensic settings.

Although not discussed in detail in our paper, narrow psychopathy dimensions are differentially related to functionally adaptive traits. There is some evidence to suggest that factor 1 traits (callous affect and manipulative interpersonal style) have a higher degree of adaptive functionality than factor 2 (antisocial behaviour and reckless lifestyle). For instance, factor 1 traits are associated with higher intellectual abilities and executive functioning than factor 2 traits (Ben-Yaacov & Glicksohn, 2018; Neumann & Hare, 2008; Pasion et al., 2018; Ross et al., 2007; Vitacco et al., 2005, 2008). Moreover, factor 1 traits can potentially protect against other mental disorders (Benning et al., 2005; Međedović et al., 2018; Ragsdale & Bedwell, 2013). Some of the studies we reviewed in Section 3 also hint at the possibility that factors 1 and 2 could have different outcomes in terms of fitness with only interpersonal traits positively predicting fertility and lifestyle–antisocial traits predicting higher morbidity. Future studies that assess how independent psychopathic facets, rather than total psychopathy scores, relate to fitness could lead to more accuracy in adaptationist models, while also acknowledging that psychopathy is multidimensional and not a uniform and categorical construct.

Finally, research on psychopathy, and particularly that concerned with evolutionary phenomena, is almost exclusively carried out on WEIRD (Western, educated, industrialised, rich and democratic) samples (Henrich et al., 2010). As a recent paper in this special issue has argued, more studies are needed to assess individual psychological differences and their fitness consequences in hunter–gatherer and other non-industrialised societies (Hunt & Jaeggi, 2022). Our prediction is that psychopathic traits would correlate with fitness in different ways across cultures. For example, in hunter–gatherer groups where individuals with dominant and anti-social behaviour are ostracised, we predict psychopathic traits to be negatively correlated with fitness. It is also worth noting that such traits may be expressed in different ways in those societies, which would require balancing emic (culture-specific) with etic (universal) approaches for measuring those traits (Chaudhary & Salali, 2022).

## 5. Conclusion

Here we reviewed the evidence for and limitations of evolutionary explanations of psychopathy. Although there is some evidence for psychopathic traits presenting adaptive trade-offs between mate-seeking and fertility on the one hand, and mortality, morbidity and decreased parental investment on the other, they do not necessarily lead to straightforward consequences on fitness. At present most research is limited to proxies of fitness rather than measurable fitness outcomes, which limits our ability to test the evolutionary predictions. A more fruitful approach would require a move away from studies that only look at the correlation between psychopathy and fitness proxies, to a more integrated approach that considers proximate mediating factors and developmental triggers. The adaptive potential of psychopathic traits should be considered and assessed in relation to the social factors such as cultural norms, which might affect whether people tolerate, ostracise or are easily deceived by psychopaths.
